# Comparative genomic analysis of novel *Acinetobacter* symbionts: A combined systems biology and genomics approach

**DOI:** 10.1038/srep29043

**Published:** 2016-07-05

**Authors:** Vipin Gupta, Shazia Haider, Utkarsh Sood, Jack A. Gilbert, Meenakshi Ramjee, Ken Forbes, Yogendra Singh, Bruno S. Lopes, Rup Lal

**Affiliations:** 1 Department of Zoology, University of Delhi, Delhi-110007, India; 2 Department of Surgery, University of Chicago, Chicago, 60637, USA; 3 School of Medicine, Medical Sciences and Nutrition, University of Aberdeen, Aberdeen, AB25 2ZD, UK

## Abstract

The increasing trend of antibiotic resistance in *Acinetobacter* drastically limits the range of therapeutic agents required to treat multidrug resistant (MDR) infections. This study focused on analysis of novel *Acinetobacter* strains using a genomics and systems biology approach. Here we used a network theory method for pathogenic and non-pathogenic *Acinetobacter* spp. to identify the key regulatory proteins (hubs) in each strain. We identified nine key regulatory proteins, guaA, guaB, rpsB, rpsI, rpsL, rpsE, rpsC, rplM and trmD, which have functional roles as hubs in a hierarchical scale-free fractal protein-protein interaction network. Two key hubs (guaA and guaB) were important for insect-associated strains, and comparative analysis identified guaA as more important than guaB due to its role in effective module regulation. rpsI played a significant role in all the novel strains, while rplM was unique to sheep-associated strains. rpsM, rpsB and rpsI were involved in the regulation of overall network topology across all *Acinetobacter* strains analyzed in this study. Future analysis will investigate whether these hubs are useful as drug targets for treating *Acinetobacter* infections.

*Acinetobacter* is a Gram negative nosocomial pathogen^1^ that causes a variety of infections in humans ranging from respiratory failure, ventilator associated pneumonia, bacteremia and wound infections^2^. The major species of *Acinetobacter* associated with nosocomial infections are *A. baumannii, A. nosocomialis, A. pittii A. johnsonii* and *A. lwoffii*^3^. Systems biology is the study of an organism, viewed as an integrated and interacting network of genes, proteins and biochemical reactions, that form the functional units capable of operations needed for cell and tissue/organ level physiological function^4^. Protein-protein interaction (PPIs) network analysis is a valuable systems biology tool for identifying drug targets and functional mechanisms^5^. PPIs can be used to elucidate the cellular events that maintain physiological stability and integrity. Using whole genome data, we have constructed protein-protein interaction networks for four strains of *Acinetobacter spp.* isolated from different animal intestines to determine the how these networks vary across environments. To delineate differences, we employed hierarchical network theory to quantify the structural properties of each network, such as the emergence of modules/communities and sparsely distributed hubs^6^,^7^, and self-organized working principle^8^. The emergence of modules/communities may correspond to independent functions obeying their own laws, with activities being nonlinear in nature^9^. The sparsely distributed hubs may interfere and control network stability within the community^9^ as well as other communities. Hubs and highly connected proteins play a crucial role in biological networks^10^.

We have sequenced and assembled the genomes of 3 *Acinetobacter spp.* strains (SFA, SFB and SFC) isolated from sheep feces, and one strain (HA) isolated from the gut of a 5^th^ instar larva of polyphagous insect, *Helicoverpa armigera*. A hierarchical protein-protein interaction network (PPI) was constructed, and subnetwork/modules analyzed, to identify regulatory proteins important for cellular physiological processes. Key proteins are defined as randomly placed, with important functional roles and a high degree of interactions^11^,^12^ in each isolated strain. The STRING v10 database for *A*. *lwoffi and A. johnsonni* was used to as a basis for building the PPI network of the four novel strains.

## Material and Methods

### Isolation and culturing of strains

*Acinetobacter* strains were isolated from sheep feces, and designated SFA, SFB and SFC. All isolates grew well at 26 °C on both Mac-Conkey agar and blood agar plates. A fourth *Acinetobacter* strain, HA, was isolated from the 5^th^ instar larva of *Helicoverpa armigera* (polyphgous pest) from an agricultural field in Maharashtra, India. *Acinetobacter* genus and species level identification was achieved by 16S rRNA gene amplification and sequencing using the universal primers 8F and 1546R, and by *rpo*B PCR and sequencing^13^.

### Whole genome sequencing and assembly

The isolated *Acinetobacter* strains were grown at 26 °C on both Mac-Conkey agar and blood agar plates until mid-log phase with shaking at 250 rpm. Whole genomic DNA extraction was performed according to the manufacturer’s instructions using the Promega Wizard Genomic DNA purification kit (Promega, Madison, WI). The concentration of DNA was determined by picogreen assay. DNA was used to construct TruSeq DNA libraries with manufacturer’s defaults, which were then sequenced on an Illumina HiSeq2000 platform with 100 base paired-end sequencing. The FASTQ paired-end reads were assembled using Velvet de-novo assembler^14^, coverage was typically 30x and assembled genome size approximately 3 Mb. Genome assemblies were validated for the misassembled and low coverage regions using BWA^15^ and Tablet^16^ software packages. Quality filtered contigs were further extended using paired-end criterion.

### Genome Annotation and Phylogenetic affiliation

Final assemblies were checked for the percentage completeness using with 31 protein encoding phylogenetic marker genes^17^, and 107 single copy marker genes^18^. Each genome revealed presence of all 31/31 and 107/107 genes, which suggests completeness. Open reading frames (ORFs) were called for each genome using FragGeneScan v1.16^19^. Predicted ORF’s were annotated by KAAS (KEGG Automatic Annotation Server)^20^ to assign KEGG orthologs (KO) identifiers to the query ORFs sequences using GHOSTXx^21^ algorithm against KEGG GENES database^22^. For automatic genome annotations, the *Acinetobacter* spp. SFA, SFB, SFC and HA genome assemblies were submitted to Rapid Annotation using Subsystems Technology (RAST) Server^23^. Annotated genomes are accessible from the RAST server by logging in with the guest account with the accession numbers (RAST-ID) 258824, 258827, 258830 & 262612 for SFA, SFB SFC and HA respectively. Assembled genomes were phylogenetically delineated using two way ANI script in PYANI Master Pipeline using percentage identity algorithm at default parameter. Reference genomes were adopted from the list of all representative species of *Acinetobacter* maintained at the Broad Institute https://olive.broadinstitute.org/collections/acinetobacter.5/strains on 14/10/2015.

### Construction of protein-protein interaction network of *Acinetobacter* strains based on phylogenetic cluster analysis

To construct protein-protein interactions for the novel strains we employed the STRING Database (v10)^24^ which is the most comprehensive *Acinetobacter* protein interaction resource. *A*. *lwoffi and A. johnsonni* were selected for PPI construction, as these were phylogenetically closer to the novel strains. The STRING v10 database consists of known and predicted PPIs, which included direct (physical) and indirect (functional) associations. Here we provided the protein sequences of novel *Acinetobacter strains* as an input and queried against *A. lwoffi* and *A. johnsonii* PPIs. The interaction associations were integrated with different sources such as genomic context, high-throughput experimental data, database and literature mining, and analysis of co-expressed genes. The PPI networks were visualized using Cytoscape Version 3.0.1^25^.

### Identification of highly regulating nodes in the network

The protein-protein interaction networks focused on finding hubs which highly connected proteins were considered to play a crucial role in biological networks. Hubs are proteins having a high degree of interactions/edges and are randomly placed in the network, having important functional roles^26^. In our study, using network analyzer, the plug-in of Cytoscape v 3.0.1 and Perl programming version 5.18.2.2, we identified that the hub proteins communicated with many other significant proteins involved in the network.

### Statistical analysis of the Network

The statistical and functional significance of the network, is proposed to be measured using various statistical parameters, namely in the proposed case, probability of degree distribution, average clustering co-efficient and average neighborhood connectivity^27^. The network is constructed to find if it obeyed power law,


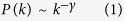


indicating the scale free nature of the network, where, γ is an order parameter which identified the different topological structure of a scale free network. The clustering co-efficient C (k), which is defined by


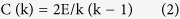


and is the ratio of the number of edges E of the node having a k degree with neighbors to the total possible number of such edges,


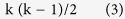


is a measure of the topological structure of the network^28^. The neighborhood connectivity of a node is the number of connected neighbors with it and characterizes the correlation pattern of connectivity of interacting nodes in the network. This connectivity correlation would be measured by defining a conditional probability


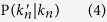


which is the probability of making a link from a node having degree k_n_ to another node of degree k′_n_^9^. Then the average neighbourhood connectivity of nodes with connectivity k_n_ is given by^9^,


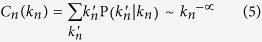


following a power law scaling behaviour with α < 1 for most of the real networks (Maslov and Sneppen, 2002; Pastor-Satorras *et al*., 2001). If C_n_(k_n_) is an increasing function of k_n_ (for negative values of α) then the topology of the network show assortive mixing^29^ where high degree (the number of edges per node) nodes have affinity to connect to other high degree nodes in the network. However,


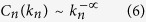


with positive values of α, is the signature of the network having hierarchical structure^29^, where low degree nodes tend to connect high degree hubs^29^ and the few high degree hubs present in the network try to control the low degree nodes. The two-centrality measurements (Betweenness centrality & Closeness centrality) were also calculated.

### The centrality measurement of the network

We considered two centrality measurements to analyze our network as described below.

#### Betweenness centrality

The betweenness centrality (C_B_) quantifies a node, occurring a number of times to bridge along the shortest path between two other nodes^6^, which could be calculated by,


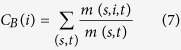


where, m (s,i,t) is the number of shortest path, connecting s and t that pass through node i; and m (s,t) is the number of shortest paths in-between nodes s and t. The sum is to be taken of all pairs (s,t) of distinct nodes. In a complex network, the nodes which have high value of C_B_ lie on paths between many other nodes, and have high influencing capability of information spreading within the network^30^.

#### Closeness centrality

Closeness centrality (C_C_) can be established in terms of “shortest path lengths” between pairs of nodes^31^. The farness of a node can be estimated by the sum of its distances to all other nodes in the network; and closeness is measured as the inverse of this farness^32^. The closeness centrality of node is defined by,


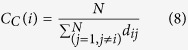


where, d_ij_ is the shortest distance between node i and j, and N is the size of the network. The C_C_ of a node in a network describes the efficiency of the node for information propagation in the network^33^. The high C_C_ valued nodes in the complex network have higher efficiency to propagate information in the entire network^34^; whereas, nodes having low C_C_ values have higher receiving capabilities of information^32^.

### Module and its functional enrichment analysis

Modules of large PPI network are defined as the set of statistics and functionally significant interacting genes^26^. MCODE^35^, the plug-in of Cytoscape, identifies the clusters that are highly interconnected regions in a network. We used default setting of MCODE, which analyzed networks, using Scoring [include loops, degree cutoff (2)] and Finding [node score cutoff (0.2), haircut, node density cutoff (0.1), K-core (2), Maximum Depth (100)] parameters that were optimized to produce the best results for the network. The potential clusters were identified by a search method, estimating their significance scores with a high score (>1) and a decent number of nodes and edges^36^. The extracted clusters were ranked by scoring through density and size. Once the nodes in a cluster were identified, one could intuitively reduce the complexity of the network by replacing the individual nodes with one large parent node, which allowed focusing on the interactions with the cluster. To understand the functional role of proteins involved in top three modules of each strain, we subjected the module proteins for GO annotation. Because modules tend to have a similar function, we over-represented the Gene Ontology categories (Molecular function, Biological process, Cellular Components) for modules in each strains network. The major categories were considered based on the percentage of each set of nodes to construct pie diagrams that allowed better visualization of the functional categories.

### Network motif

In biological networks, these motifs are suggested to be recurring circuit elements that carry out key information processing tasks^37^. To understand these complex networks, we sought to break down such networks into basic building blocks. A network motif was defined based on the criterion that the number of occurrences must be at least five, and also must be significantly higher than that used in randomized networks. We applied FANMOD^38^ on the complete network, to select network motifs. The significance test was carried out on 1000 randomized networks, and a pattern with P < 0.05 was considered statistically significant. Clusters were analyzed for three node motif, using MCODE, from which we identified the motif within highly clustered nodes.

## Results

### Whole genome sequencing and phylogenomic analysis of *Acinetobacter* strains

**Figure 1:**
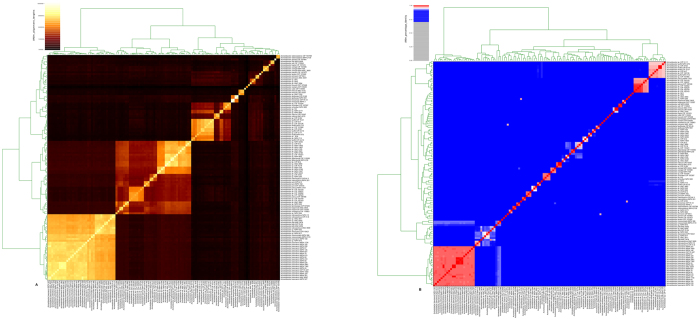
Dual dendrogram of novel *Acinetobacter* strains (**A**) Genome to genome alignment coverage (%). (**B**) Genome-genome similarity identity (%) of SFA, SFB, SFC and HA with respect to 119 reference genotypes. Color bar predicted correlation coefficients (0 to 1.0) are shown with the color scale on the basis of respective percentage identity.

Approximately 1 Gbp of sequence was generated for each *Acinetobacter* strain (SFA, SFB, SFC and HA). Draft genomes were de novo assembled - SFA (25 contigs), SFB (26 contigs), SFC (121 contigs) and HA (102 contigs) - with an average genome size of 3.0 Mb and the average G+C content was 40% (Table 1). Two way ANI using genome similarity identity % demarcated *A. lwoffi* as the closest relative for SFA (identity % 89), while SFB and SFC were closer to *A. johnssonii* (83% & 84% identity, respectively). The HA strain, isolated from insect gut, was most closely related to *A. schindleri* (97% identity; Fig. 1). Therefore, SFA, SFB and SFC were most closely related to generally non-pathogenic or opportunistic pathogens such as *A. lwoffi* and *A. johnssonii*, which have previously been isolated from bacon, eggs, fish, and frozen food, and show resistance to desiccation and disinfectants^39^. *A. lwoffi* is commonly associated with human skin, but has been linked to bacteremia, cancer and systemic lupus erythematosus^40^. *A. johnssonii* is mostly associated with the environment and has occasionally been linked to infections^41^. *A. schindleri* has been suggested to be a misidentified opportunistic pathogen in patients with underlying predisposition^42^.

### Characterization of protein-protein interaction network in four *Acinetobacter* strains

**Figure 2:**
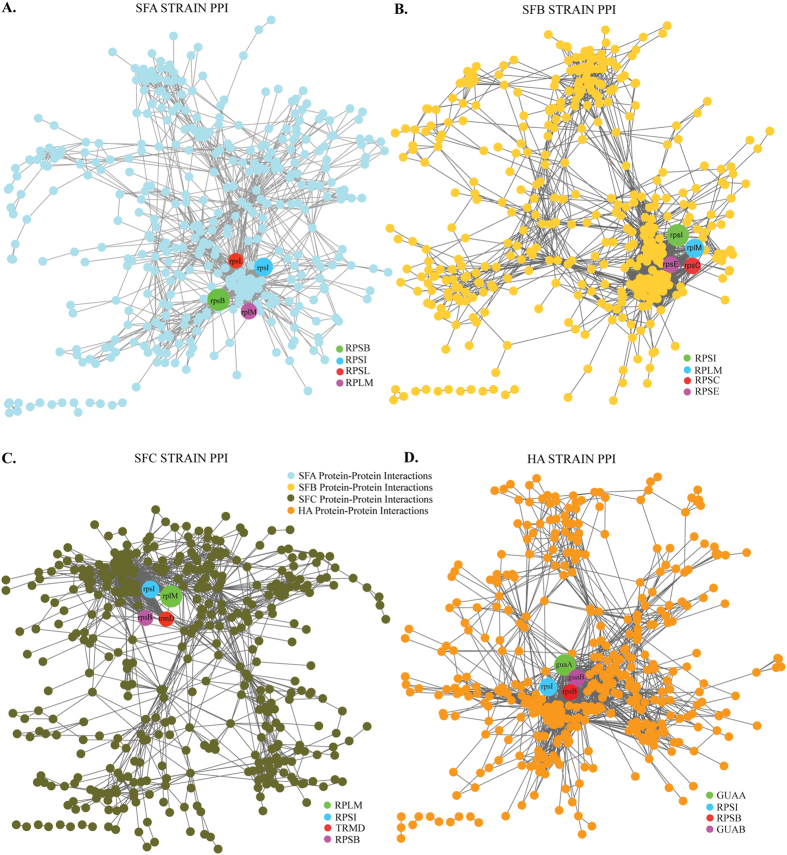
The PPI network of four novel *Acinetobacter* strains. Expanded view of the network imported from Cytoscape, where nodes represent proteins and edges the physical interaction. **(A)** All nodes and edges of SFA strain PPI are filled circles (cyan) and lines (gray), respectively. **(B)** All nodes and edges of SFB strain PPI are filled circles (yellow) and lines (gray), respectively. **(C)** All nodes and edges of SFC strain PPI are filled circles (green) and lines (gray), respectively. **(D)** All nodes and edges of HA strain PPI is filled circles (orange) and lines (gray), respectively.

Cataloguing the stable and transient PPIs in a cell can facilitate functional annotation of gene products, providing insights into the organization of the proteome. Following removal of redundant interactions and protein nodes, the resulting network had 2693 interactions, involving 422 proteins for SFA, 2620 interactions involving 414 proteins for SFB, 2401 interactions involving 426 proteins for SFC, and 2638 interactions involving 420 proteins for HA (Fig. 2).

**Figure 3:**
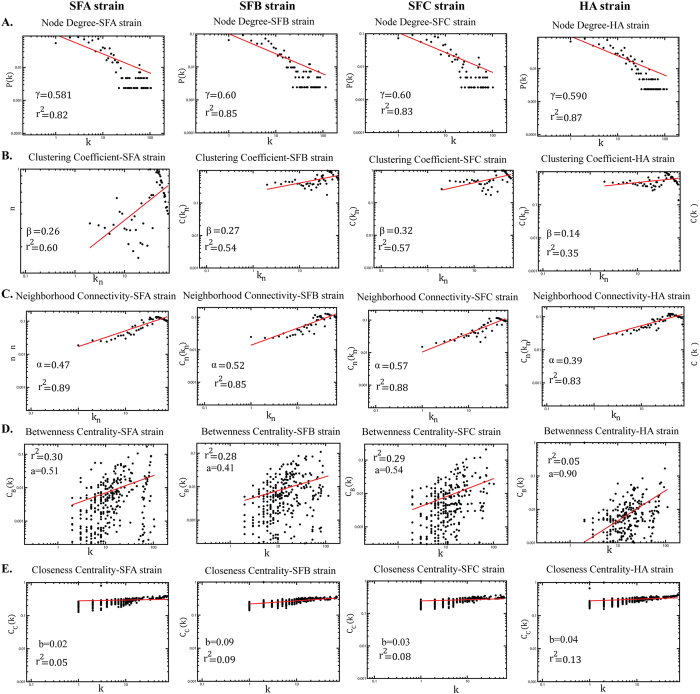
The topological properties of four novel *Acinetobacter* strains network depicting with correlation coefficient values (r2): **(A)** probability of degree distribution P(k), **(B)** average clustering coefficient, **(C)** average neighborhood connectivity, **(D)** Betweenness centrality and **(E)** Closeness centrality of the PPI network. All these properties follow the power law distribution and show the nature of scale-free network, suggesting a hierarchical organization in the network.

The topological properties of each PPI network were parameterized with probability of degree distribution P (k), which suggested that each network followed a power law scaling behavior


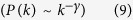


with the value of the degree exponent γ ~ 0.6 in all the four strains (Fig. 3A–D). A straight-line fitted to the data curve with a correlation co-efficient value of ~0.8 in all the four stains. The small value of γ (γ < 2) indicated that the network was hierarchical^8^, signifying the emergence of hierarchical modules and/or communities^7^, with a sparse distribution of highly connected hubs. That the few highly-connected hubs are connected to many low-degree nodes is indicative of a regulatory power of the hubs over these nodes. In confirmation of a hierarchical network^7^, the average clustering co-efficient C(k_n_), calculated as a function of number of neighbors k_n_, again followed the power scaling law given by


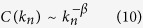


with β = ~0.1 in all the four strains. The straight line is the fitted curve with correlation coefficient value of ~0.5 in all these four strains (Fig. 3A–D).

Average neighborhood connectivity C_n_ (k_n_), constructed as a function of k_n,_ also followed a power scaling law given by,


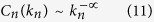


with α = ~0.5 for all the four strains (Fig. 3A–D), also supporting that the network is hierarchical^7^, where the straight line is the fitted curve with correlation coefficient of ~0.8 to the data points. The number of degree (edges per node) was calculated based on each PPI network, and we list here the main hubs for each network with their degrees: rpsB (106), rpsI (105), rplm (103) *and* rpsL (90) (SFA - Fig. 2A), rpsI (116), rplm (114), rpsC (87) *and* rpsE (87) (SFB Fig. 2B), rplm (104), rpsI (102), rpsB (82) *and* trmD (78) (SFC Fig. 2C) and guaA (148), rpsI (110), guaB (97) *and* rpsB (93) (HA Fig. 2D). The four genes in each strain network are likely indicative of key regulatory functions in each genome^33^. The modular topological structure of the network demonstrated the existence of various functional modules or sub-networks, and also the organization among these modules^7^.

For the networks, C_B_ and C_C_ followed power law scaling behavior with *k*, 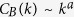, and 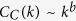, where a and b are positive values (Fig. 3D,E). It was also found that the fitted straight lines on the network data of these two centrality measurements were approximately parallel with the average value of “a” equal to 0.51 (SFA), 0.41 (SFB) 0.54 (SFC) and 0.90 (HA), and “b” equal to 0.02 (SFA), 0.09 (SFB) 0.03 (SFC) and 0.04 (HA). The increasing value of C_B_ with *k* indicated that high degree nodes have the greatest information spreading capability in the network. Further, C_C_ analysis shows that high degree nodes rapidly disseminate signal information to low degree nodes. Therefore, the four hubs in each PPI network are the main signal propagating nodes in both the network, and their respective modules.

### Modules and functional enrichment analysis

**Figure 4:**
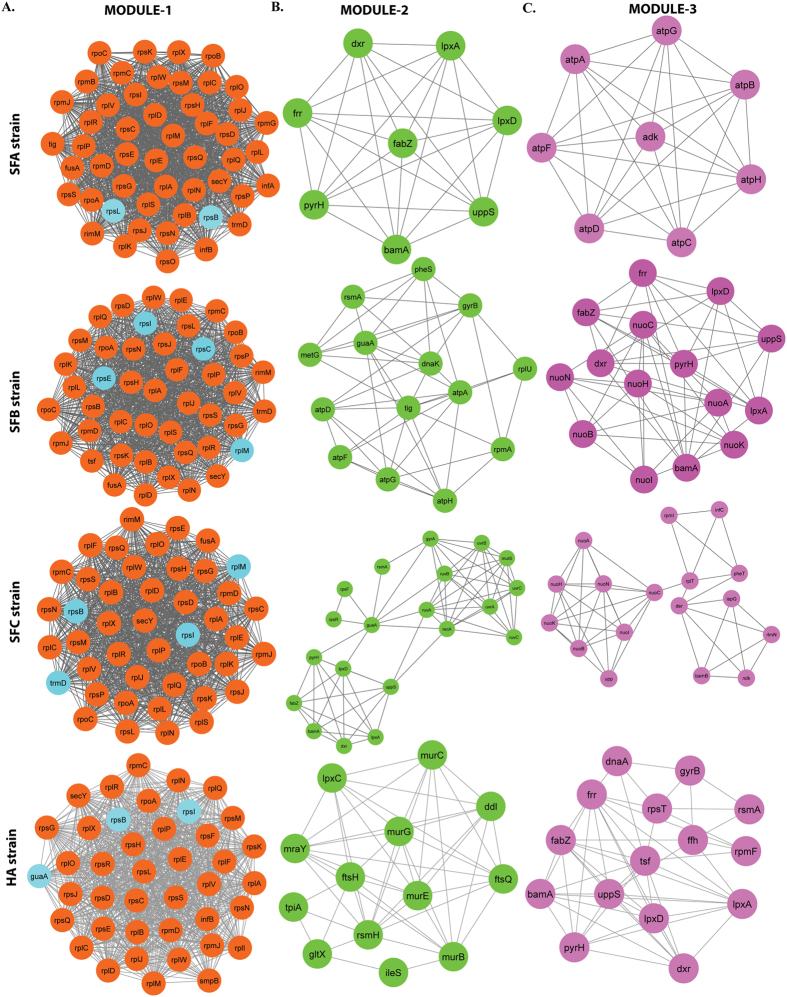
Skeletal structure of the modules in the novel *Acinetobacter* strains PPI network. All the modules 1–3 are constructed and analyzed using MCODE. **(A)** In module 1 all the nodes are in filled circles (orange), with scoring value 48.85 (SFA), 44.54 (SFB), 43.62 (SFC) and 39.65 (HA); **(B)** modules 2 all the nodes are in filled circles (green), with scoring value 8 (SFA), 7.38 (SFB), 7.15 (SFC) and 8 (HA), **(C)** module 3 all the nodes are in filled circles (pink), with scoring value 8 (SFA), 7.14 (SFB), 5.62 (SFC) and 7.71 (HA) with the corresponding edges in grey lines.

**Figure 5:**
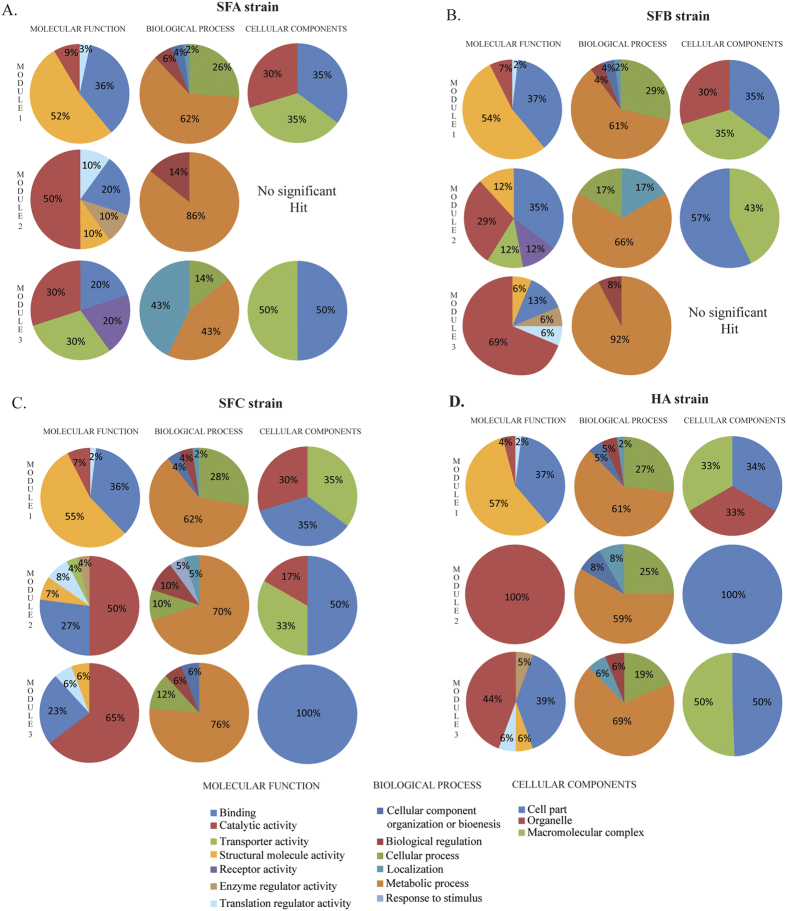
Functional annotation of three modules depicting biological processes, cellular component and molecular function represented in pie charts. **(A)** Modules of PPI in HA strains. **(B)** Modules of PPI in strain SFA. **(C)** Modules of PPI in strain SFB. **(D)** Modules of PPI in strain SFC.

We identified significant modules (Fig. 4) in each network that could have distinct biological functions, and were functionally separable. Three such significant clusters were identified for each strain (SFA, SFB, SFC and AHA; Fig. 4A–C). Each module was ranked based on the MCODE network score^43^. The details of each module were represented in Table 2. Module 1 of SFB and SFC includes four of its hubs (SFB: rpsI, rplM, rpsE, rpsC *&* SFC: rplM, rpsI, rpsB and trmD), while HA has three hubs (guaA, rpsB and rpsI), and SFA had two hubs (rpsL and rpsB; Fig. 4A). This indicated that most of the significantly large hubs did not only interfere in the internal regulation of their own modules in the network, but also affected other modules. However, hubs like trmD (SFC), guaA, and guaB (HA) were not present in any of the other modules, suggesting that these hubs indirectly interfered with modular properties and activities. These modules were found to be linked via sparsely distributed nodes, which can mediate cross talk among the modules^44^. Functional enrichment analysis for molecular, biological and cellular components was performed in the three modules for each strain (Fig. 5A–D). Module proteins were performing similar functions in each strain isolated from sheep. Module-1 of all strains was majorly involved in structural molecular activity, whereas module-2 and 3 were involved in catalytic activity. Metabolic pathway is the common and highly involved biological process by all the proteins present in each module of all the four strains.

### Network Motif

**Figure 6:**
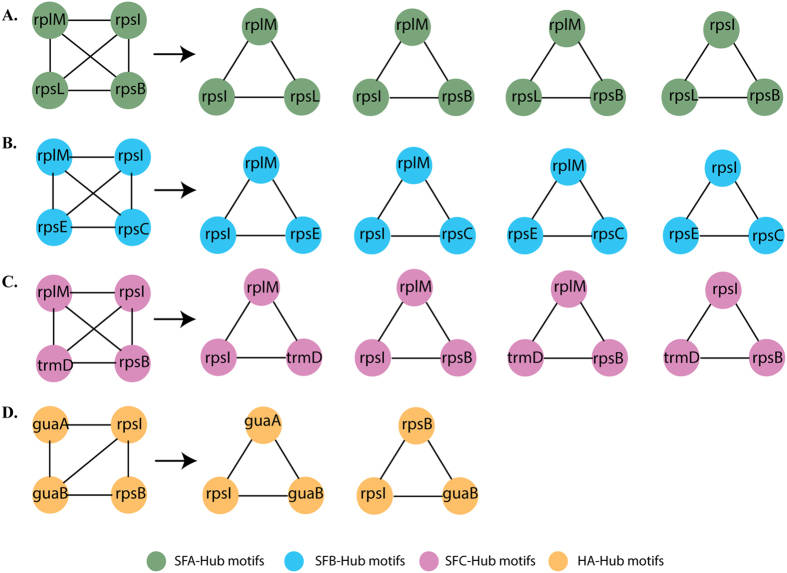
Hub motifs in the *Acinetobacter* strains PPI network. **(A)** Hub-node motifs of SFA strains showing four motifs (green); **(B)** Hub-node motifs of SFB strains showing four motifs (blue); **(C)** Hub-node motifs of SFC strains showing four motifs (pink); **(D)** Hub-node motifs of HA strains showing two motifs (orange).

A statistically significant basic skeleton of three node motifs were identified with frequencies of 46%, 22%, 44%, 30% (P < 0.05) in SFA, SFB, SFC and HA, respectively (Fig. 6A–D). This motif appeared in at least 5 out of 1000 random permutations of the PPI networks (FANMOD).

MCODE was used to further identify additional significant motifs in the four PPI networks, and 3 node motifs were found in all four strains, whereas four node motifs were only identified in SFB network. We also found that hubs nodes were interacting with each other and forming a three-node motif, which was similar to the pattern of motif identified by FANMOD. The HA hub node motif interaction was different to the other strains. In SFA, SFB and SFC all the hubs were interacting with each other (total six edges in each strain) (Fig. 6A–C); whereas HA hubs protein were interacting each other with possible five edges (Fig. 6D). The four hub motifs in HA were further classified into 2 different three-node motifs (Fig. 6D), whereas in SFA, SFB and SFC the four hub node motifs were further classified into 4 different three-node motifs (Fig. 6A–C).

## Discussion

Understanding signaling processes and identifying interacting proteins could be essential in identifying novel drug targets in *Acinetobacter*. Mathematical models such as Bayesian networks, ordinary differential equations, boolean network and Petri nets have previously been used to try and pinpoint proteins that are important in networks^45^,^46^. The network-based approach applied here, using experimentally observed and literature-available data, enables the construction of preliminary models to understand system regulation in *Acinetobacter*. Each module/sub-network was cohesive to the sub-subnetwork as reported earlier^47^, and each sub-subnetwork (module) was reduced to a cluster of connected triangles (basic motifs), corresponding to significant positive and negative feedback loops^47^. Further we found that each network comprised three smaller modules, with different functional components. The three modules in each network exerted the most influence over the main regulating network, with the main hubs acting as rapid signal dissemination nodes throughout the functional modules. These main hubs were mostly found in the main module-1, because it had the capacity to influence the other modules. Further splitting modules to smaller level i.e. hubs which are seed genes of the whole interacting network, we identified nine genes as important network regulators (*guaA*, *rpsI*, *rpsB*, *guaB*, *rpsL*, *rplM*, *rpsC*, *rpsE* and *trmD*), where *rpsI* was found to be important as it was present in all the four strains. Identified hubs genes were mainly associated to the translation process. The genes *rpsB*, *rpsI*, *rpsL*, *rpsE* and *rpsC* encode the bacterial ribosome protein 30S subunit (small ribosomal subunit of prokaryotic system), whereas *rplM* is encodes the 50S (large ribosomal subunit). The *guaA* encodes for GMP synthase (EC: 6.3.5.2) whereas *guaB* encodes an IMP dehydrogenase (EC: 1.1.1.205). Our studies indicate that the GuaA and GuaB proteins are critical for the survival of bacteria and could play an important role in the infection cycle of *Acinetobacter*, as shown earlier in the case of tick borne pathogen *B. burgdorferi*^48^ Human IMP dehydrogenase inhibitors are validated targets for immunosuppressive, antiviral and anticancer drugs, but the potential of microbial IMP dehydrogenase inhibitors has yet to be exploited in antimicrobial chemotherapy^49^. The *trmD* gene which is essential for bacterial growth is a tRNA modification enzyme encoding the enzyme tRNA (guanine^37^-N^1^)-methyltransferase D (EC 2.1.1.228) responsible for converting G37 to m(1)G37 on the 3′ side of the tRNA anticodon. This enzyme is responsible for one carbon group methyl transferase^50^. More specifically for the transfer of the N_1_-methyl group on the upcoming tRNA from S-adenosyl-L-methionine^51^. This methylates the methionine which is the first amino acid in translation step. The reaction involves methyl transfer from S-adenosyl methionine and is critical to minimize tRNA frameshift errors on the ribosome.

The network for strain SFA comprised 4 hubs (Fig. 6A) with one large ribosomal rplM, which is largest of all subunits (L13) along with the three smaller ribosomal subunits rpsI, rps and rpsB. Strain SFB motif network was including the almost same hubs (Fig. 6B) with large subunit, rplM (L13) along with the smaller subunits rpsI, rpsE and rpsC. Their close interaction indicates the predominance of translational process for the cell system maintenance. Another gut isolate, SFC shared the same hubs (Fig. 6C) of large and small subunits of ribosomal assembly along with trmD. The insect gut isolate, strain HA comprised of two smaller subunits of ribosomal assembly motifs rpsI and rpsB, along with two other hubs guaA and gubB (Fig. 6D). The genes that encoded them are involved in de-novo purine biosynthesis and its metabolism. They carry out the two step reaction involving the conversion of inosine monophosphate (IMP) to xanthosine monophosphate (XMP) by inosine 5′-monophosphate dehydrogenase^44^ coded by gene guaB. Second step is the conversion of the XMP to guanosine monophosphate (GMP) by Guanosine monophosphate synthetase coded by *guaA*^48^. The first step is the rate limiting step which ultimately determines the denovo synthesis of guanine nucleotide whereas the second step is the branching point of pathway where synthesis of guanosine or adenine is diverged in de novo purine synthesis. The motif analysis demonstrated that the interaction between rplM, rpsB and rpsI was crucial, with a regulatory function in SFA and SFC. The *rpsI* gene is present in all *Acinetobacter* strains as a regulatory gene. Even *guaB* in the HA network, which was not present in any of the three modules, acted as a mediator to cross talk among the modules and also indirectly interfered with modular properties and activities. Network motif analysis also suggests *guaA* and *guaB* are key regulatory components for the pathogenic strains of *Acinetobacter* spp.

On the basis of whole genome phylogeny we propose the names *Acinetobacter aries* sp. nov. for strain SFA, *Acinetobacter ovis* sp. nov. for the strains SFB and SFC and *Acinetobacter armigera* sp. nov. for strain HA. Although, in silico predictions warrant further experimental confirmation of the key regulators, the current study lays the foundation in order to understand the role of key regulators in *Acinetobacter*. Targeting functional genes involved in regulation of hierarchical protein networks provide us with an alternative way in treating infections caused by *Acinetobacter* spp.

## Additional Information

**Accession codes**: The accession numbers for the Acinetobacter sp. of SFA, SFB SFC and HA were LSZI00000000, LSZH00000000, LSZG00000000 & AJXD00000000 respectively.

**How to cite this article**: Gupta, V. *et al*. Comparative genomic analysis of novel *Acinetobacter* symbionts: A combined systems biology and genomics approach. *Sci. Rep.* **6**, 29043; doi: 10.1038/srep29043 (2016).

## Figures and Tables

**Table 1:** General genomic features of *Acinetobacter* strains for comparative analysis.

Genome	Estimated genome size	Average GC content	CDS	Coding density	Total rRNA	tRNA	rRNA	Status	Source	Accession No.
*Acinetobacter* sp SFA	3.13	42	3140	86.38	80	74	6	Draft	Sheep	LSZI00000000
*Acinetobacter* sp SFB	3.30	38	3231	85.11	72	68	4	Draft	Sheep	LSZH00000000
*Acinetobacter* sp SFC	3.32	38	3268	85.52	75	69	6	Draft	Sheep	LSZG00000000
*Acinetobacter* sp HA	3.12	41	3140	85.91	77	64	13	Draft	Insect	AJXD00000000

**Table 2:** Modules in four novel *Acinetobacter* strains.

Strains	Module-1	Module-2	Module-3
SFA-Nodes	50	8	8
SFA-Edges	1197	28	28
SFA-Scores	48.857	8	8
SFB-Nodes	45	14	15
SFB-Edges	980	48	50
SFB-Scores	44.545	7.385	7.143
SFC-Nodes	44	20	17
SFC-Edges	938	68	45
SFC-Scores	43.628	7.158	5.625
HA-Nodes	41	13	15
HA-Edges	793	48	54
HA-Scores	39.65	8	7.714
